# Innovative Data-Driven Approaches to Community Health and Prosperity: Lessons From the City of Lauderhill’s Engagement and Improvement Strategy

**DOI:** 10.7759/cureus.97800

**Published:** 2025-11-25

**Authors:** Farzanna Haffizulla, Siya Khanna, Luis F Corona, Anam Ahmed, Melissa P Dunn, Patrick Hardigan

**Affiliations:** 1 Internal Medicine, Nova Southeastern University Dr. Kiran C. Patel College of Osteopathic Medicine, Fort Lauderdale, USA; 2 Osteopathic Medicine, Nova Southeastern University Dr. Kiran C. Patel College of Osteopathic Medicine, Fort Lauderdale, USA; 3 Miller School of Medicine, University of Miami, Miami, USA; 4 College of Business, Florida International University, Lauderhill , USA; 5 College of Health Sciences, University of Wyoming, Laramie, USA

**Keywords:** caribbean, collective impact, cultural humility, diverse, health disparities, health equity, intersectionality, partnership, social determinants, underserved

## Abstract

Research demonstrates that social vulnerability has a disproportionate effect on the utilization of health care services among minority populations. The City of Lauderhill (CoL) is predominantly composed of racial/ethnic minorities, including a significant proportion of Caribbean-born and foreign-born residents, particularly from Jamaica and Haiti. CoL has one of the highest social vulnerability indices in the US and is designated by the US Housing & Urban Development (HUD) as a low-income area. The COVID-19 pandemic and existing community challenges set the stage for forming The Lauderhill Health & Prosperity Partnership (LHPP), which utilized a collaborative, multipronged approach to address the social determinants of health (SDOH). This included a community needs assessment (CNA) and Asset-Based Community Development (ABCD) grounded in Collective Impact 3.0 (CI), which leveraged a partnership with Nova Southeastern University as an academic partner and anchor institution. Population health resident data outlined in the CNA leveraged NSU’s community-based participatory research platform of the Caribbean Diaspora Healthy Nutrition Outreach Project (CDHNOP) to gain valuable insight into relevant SDOH and health metrics. CDHNOP employs trusted disease prevention-focused culturally tailored health messaging through social media, health programming, and other avenues.

Our specific aims were to quantify SDOH and health metrics most relevant to this diverse population and engage public and private partnerships while harnessing city residents' collective input and involvement to engage in data-driven city-wide improvements in SDOH domains. Qualitative focus group data and quantitative surveys were used in this study. A needs assessment questionnaire developed using the Protocol for Responding to and Assessing Patients' Assets, Risks, and Experiences (PRAPARE) tool was paired with existing public and city data to provide a comprehensive assessment.

Findings revealed significant challenges in social and economic vulnerability within the city, with an alarming 18-fold increase in homelessness from 2020 to 2021. A total of 235 residents partially or fully completed the survey. Among respondents, 44% reported housing insecurity, 22% were unemployed, and 16% experienced food insecurity. Focus group themes emphasized barriers to healthcare, systemic distrust, and gaps in mental health access. Survey data revealed high social vulnerability scores aligning with elevated self-reported health burdens (e.g., asthma, hypertension), reflecting systemic inequities. Higher rates of overall social vulnerability were particularly pronounced in the southeast region of the city, mirroring rates of diabetes, asthma, poor physical and mental health, and smoking. Mental health, well-being of children, access to healthcare, lack of prevention and nutrition initiatives, and distrust of the healthcare system also arose as issues. These results point to a need for place-based, equity-focused interventions that align with broader national health equity frameworks, particularly for similarly diverse urban communities.

## Introduction

The rationale for this study is based on the urgent need for localized social determinants of health (SDOH) assessments in racially and socioeconomically diverse cities like Lauderhill, USA, to inform equitable public health planning. Lauderhill’s demographic profile, characterized by high proportions of Black and Caribbean residents and elevated poverty levels, underscores the city’s relevance as a case study for place-based SDOH strategy development. The City of Lauderhill includes 8.5 square miles of Broward County, Florida, and 8,422 individuals per square mile. As of 2020, there are 15 census tracts with a total population of approximately 74,482, of which 85% are racial/ethnic minorities [1]. The city, designated by the US Housing & Urban Development (HUD) as low-income, contains two opportunity zones, has the second-highest density in Broward County, and is 98% built out. 

The demographics of the City of Lauderhill make it a unique and diverse community, representing a population of varying ages and ethnic/racial backgrounds. The median age in Lauderhill is 36.2, with 58% of the population between 18 and 64 years of age, 26.8% under 18 years of age, and 14.9% 65 years of age or older. The city comprises 40,533 women, accounting for 56% of the population, and 31,330 men, approximately 43.6% of the city’s residents [1]. 

The City of Lauderhill has a 37.3% foreign-born population, with 95% originating from Latin and Central America, as well as Island nations in the Caribbean. Specifically, this includes 82.9% from the Caribbean (Jamaica 44.1% and Haiti 30%), 8% from South America (2.7% Colombia, 1.7% Peru, and 1.1% Venezuela), and 1.6% from Central America [1]. Despite its distinct demographic profile and structural challenges, Lauderhill has limited city-specific SDOH data available to inform policy or resource allocation. This study fills that gap through a mixed-methods analysis grounded in local partnerships and a resident-centered framework. This study addresses a critical gap in localized SDOH data within mid-sized, majority-minority cities. Given Lauderhill’s high social vulnerability, a granular understanding of residents’ lived experiences is essential to inform targeted policy and programmatic responses. 

Social vulnerability refers to the negative impact on a community due to external stressors such as poverty, crowded housing, and lack of accessibility to transportation or medication. Previous research suggests that social vulnerability is associated with disparity in the utilization of health care services amongst minority populations. As the City of Lauderhill has a large minority population, the rate of overall social vulnerability, measured by the social vulnerability index (SVI), within the City of Lauderhill (0.76) is approximately 31% higher than in Broward County (0.58), which is highly significant for most regions within the city [2]. Only a few regions within the city's southwest region have SVI rates in the lower quintiles. Higher rates of overall social vulnerability appear to be most pronounced in the southeast region of the city, similar to rates of diabetes, asthma, poor physical and mental health, and smoking. 

In response to existing community challenges and the burden brought on by the COVID-19 pandemic, the Lauderhill Health & Prosperity Partnership (LHPP) was developed [2]. The LHPP utilized a collaborative approach to address the social determinants of health (SDOH) of Lauderhill residents. This approach included completing a community needs assessment and Asset-Based Community Development (ABCD) grounded in Collective Impact 3.0 (CI), which leveraged an academic partnership with Nova Southeastern University as an anchor institution. Collective Impact 3.0 emphasizes authentic resident participation, shared vision development, and a focus on structural change rather than isolated interventions [3]. CI is a movement-building paradigm that utilizes authentic community engagement to bring together a diverse group of stakeholders, including residents, to co-create solutions for their community using high leverage and systems focus grounded in relationships and trust [2].

ABCD is an asset-based, locally focused, and relationship-driven process that uses the capacities (assets) of local people (residents who call the neighborhood home) and their association to build powerful communities. It is a strategy to identify available assets within the community, connect and engage with residents, and use their talents to help solve their problems and build a better community. In its unique and novel approach, the LHPP creates an inclusive participatory process of individuals, groups, and organizations from all backgrounds in the community to discuss ideas and concerns, identify resources, and develop interventions to address SDOH, including education access and quality, healthcare access and quality, economic stability, social and community context, and neighborhood and built environment. Using this framework, LHPP works to strategically design policy, environment, and systems interventions to transform Lauderhill into a healthier and more prosperous community, while implementing best practices to eliminate health disparities. 

The organizational structure includes leadership and co-created solutions by city government officials, residents, and community partners, with each LHPP sub-council focused on SDOH and related domains: health access and quality, education access and quality, social and community context, economic stability, neighborhood, and built environment, communications, and research. The LHPP is focused on ultimately improving the health outcomes, economic prosperity, environmental, and safety needs of those living and working within the City of Lauderhill. The City of Lauderhill is committed to transforming its community into a healthier, safer, and more prosperous municipality that will continue to expand and grow [2]. 

It is with this growth and transformation that city officials sought to understand the health, economic, environmental, safety dynamics, and disparities occurring within the city limits to implement necessary changes and select a model based on best practices to develop solutions to any challenges. Due to an unmet need within the City of Lauderhill to understand the complexities of health, economic, social, geographic, and life factors driving prosperity, well-being, and quality of life within the city, a community needs assessment questionnaire was disseminated with the goal of further understanding and quantifying demographics, health indicators, lifestyle, and social determinants of health. In partnership with Nova Southeastern University, the Caribbean Diaspora Healthy Nutrition Outreach Project (CDHNOP), a robust population health platform, captured population health resident data [4]. The CDHNOP uses trusted, culturally tailored health messaging through social media, print, radio, community health programs such as “In the Kitchen with Dr. H'' and digital media to promote disease prevention among Caribbean Diaspora in the US. 

Using this data, a comprehensive community assessment tool was created. This survey included questions from Protocol for Responding to and Assessing Patients' Assets, Risks, and Experiences (PRAPARE), a diabetes risk index from the American Diabetes Association, and questions related to health screening, diagnosis, and disease-associated challenges. PRAPARE is a tool developed to help health providers assess and identify social determinants of health within a community, driving certain health outcomes and behaviors [5]. This data allows healthcare providers to further understand the increased complexity of their patients and thus transform the care they provide using an integrated approach. The social determinants of health questions used were derived from the PRAPARE assessment. PRAPARE dimensions have been condensed into meaningful clusters, with studies demonstrating significant associations between higher social risk cluster scores and poor hypertension and diabetes control (acylated hemoglobin (HbA1c) ≥ 9% and BP ≥ 140/90 mm Hg) [6,7]. Addressing social determinants of health and barriers may help to improve a community's health outcomes. The rationale for this study is based on the urgent need for localized SDOH assessments in racially and socioeconomically diverse cities like Lauderhill to inform equitable public health planning. 

Our efforts aimed to quantify the SDOH and health metrics most relevant to the City of Lauderhill population. While employing city residents' collective input and involvement, we sought to engage public and private partnerships to achieve data-driven city-wide improvements in the SDOH. 

## Materials and methods

Study participants and questionnaire

Participants were recruited to take part in a one-time, anonymous survey approximately 15 minutes in length. To be eligible for this study, participants had to be 18 years of age or older and either be a resident of the City of Lauderhill and/or work in the City of Lauderhill (Table 1). Inclusion criteria required participants to be Lauderhill residents and/or those employed in the city, aged 18 or older. Incomplete responses were excluded listwise from relevant analyses. Data was collected on a secure web-based Research Electronic Data Capture (REDCap) survey platform. Integrating PRAPARE into electronic health records has been shown to be feasible across centers, with effective workflow integration and demonstrated utility in guiding care initiatives [8]. Utilizing the Institutional Review Board (IRB)-approved CDHNOP population health platform, a comprehensive community survey and focus group procedures were created for the City of Lauderhill. All surveys and focus group methods were approved through Nova Southeastern University’s IRB. The approved survey was widely disseminated through the City of Lauderhill communication networks such as neighborhood associations, community centers, local live and virtual events, direct marketing, places of worship, and social media platforms. Surveys were anonymized and included questions on demographics, health indicators, lifestyle, and social determinants of health. 

**Table 1:** Inclusion and exclusion criteria of study participants

Inclusion criteria	Exclusion criteria
Work or reside in the City of Lauderhill	Unable to read or comprehend the survey contents
Age 18 or older	Children under 18
Read, speak, write and understand English	Limited access to technology that allows for survey access/completion

Quantitative data acquisition

Early in the City of Lauderhill comprehensive needs assessment (CNA) process, the Broward Regional Health Planning Council was engaged to garner available public data relevant to the City of Lauderhill, focusing on health metrics and SDOH. A data report was prepared by FHEED, LLC (Food for Health, the Environment, Economy, and Democracy) for the Broward Regional Health Planning Council (BRHPC) based on the 2020 release of the PLACES Project, with 2018 data from the Centers for Disease Control and Prevention (CDC). In addition, a City of Lauderhill community questionnaire was created through the Nova Southeastern University (NSU)’s Dr. Kiran C. Patel College of Osteopathic Medicine (KPCOM) Department of Internal Medicine’s institutional review board (IRB) approved health equity research platform, Caribbean Diaspora Healthy Nutrition Outreach Project. All research approvals were obtained in accordance with IRB policies and human subjects research protection regulations. Other quantitative data sets were obtained in partnership and data sharing with the City of Lauderhill Police Department, and Fire Rescue/emergency medical service (EMS). The City of Lauderhill Police Department’s Records include Criminal, non-criminal, & Domestic incident reports. The data from the City of Lauderhill Fire Department includes all medical calls by primary impressions and the transport hospital that was used [2]. 

Qualitative data acquisition

Qualitative methods were used to obtain data about individuals in the community and further understand their needs. Focus group participants were identified in collaboration with city officials, residents, and community leaders to include representatives from healthcare, education, nonprofit organizations, youth advocates, business leaders, senior groups, and faith-based institutions. All focus group notes were typed during each session, and all participants remained anonymous. Each session lasted approximately 60 minutes. We conducted a qualitative analysis using a descriptive, case-based approach that prioritized the preservation of individual participant perspectives. Asset-Based Community Development (ABCD) models have been effective in enhancing community engagement and amplifying resident capacities for health improvement projects [9]. Rather than applying formal coding procedures, we utilized a thematic summary method in which each participant’s responses were reviewed in full and key insights were extracted through holistic reading and reflective interpretation. These insights were then organized into summary matrices that aligned with core topics explored in the focus groups. This approach ensured that all participant viewpoints were represented and enabled the identification of patterns and divergences without fragmenting narratives into discrete codes. The analysis was guided by immersion in the data, iterative discussion among the research team, and a commitment to maintaining the integrity of each participant's voice. This non-coding approach aligns with qualitative traditions such as immersion/crystallization and case-centered thematic description, which emphasize rich, contextual understanding over categorical abstraction. In addition, this descriptive, case-based analysis approach was selected to prioritize the integrity of individual narratives over reductionist coding and aligns with interpretive qualitative methodologies where depth and context supersede coding frequency. 

Key questions were posed to participants to solicit their views on various aspects, including the city's strengths, challenges, threats, opportunities, and potential areas for improvement, particularly related to social determinants of health and community well-being. Focus groups were held to identify positive and negative factors affecting residents and those working within the city and the overall community. The focus groups included a total of 67 participants who varied in professions and were representative of varying entities and industries. A total of eight focus groups were held and represented business owners (11 participants), faith leaders (five participants), residents (10 participants), healthcare (10 participants), non-profits (seven participants), educators (four participants), seniors (12 participants), and youth advocates (eight participants). 

Statistical analysis

Summary statistics are calculated for all study variables. In the reported results, the dominator is the number of returned surveys. Given the descriptive nature of the study, we report percentages with one decimal place. The Fisher-Exact test was used to examine the relationship between COVID-19 diagnosis and respondents' demographic characteristics. R 4.2.1 was used for the data analysis, and statistical significance is reported at p < 0.05.

## Results

Quantitative data results

Demographics

A total of 235 participants completed the survey in full or in part. Of note, the volume of missing data in the survey may have introduced nonresponse bias, limiting the precision and representativeness of descriptive statistics. Demographic results reveal that the median age was 51 years (range = 72 years), 157 (67%) were female, 39 (17%) identified as African American and 11 (5%) as Hispanic, respectively, 127 (54%) are native to the US, 110 (47%) native language is English, and the median household composition is two individuals (Table 2). 

**Table 2:** Demographic characteristics

	Overall (N=235)
Sex	
Female	157 (66.8%)
Male	52 (22.1%)
Other	5 (2.1%)
Missing	21 (8.9%)
What is your country of origin?	
USA	127 (54.0%)
Caribbean	55 (23.4%)
Other	18 (7.7%)
Missing	35 (14.9%)
Primary Language	
English	110 (46.8%)
Unknown	1 (0.4%)
Missing	124 (52.8%)
Ethnicity	
Not Hispanic or Latino	102 (43.4%)
Hispanic or Latino	11 (4.7%)
Missing	122 (51.9%)
Race	
African American	39 (16.6%)
Afro-Caribbean	13 (5.5%)
American Indian	1 (0.4%)
Asian	2 (0.9%)
West Indian	10 (4.3%)
White	29 (12.3%)
Multiple Races	12 (5.1%)
Other	4 (1.7%)
Missing	125 (53.2%)
Age	
Mean (SD)	53.7 (14.5)
Median [Min, Max]	51.0 [18.0, 90.0]
Missing	67 (28.5%)
Number of Family Members Currently Living With	
Mean (SD)	2.59 (1.62)
Median [Min, Max]	2.00 [1.00, 10.0]
Missing	136 (57.9%)

Regarding socio-economic conditions, 94 (40%) had schooling beyond high school, 62 (26%) were working full-time, 50 (21%) worked 35-59 hours per week, and 56 (23%) held one job (Table 3). These findings suggest economic instability exceeds county-wide averages, indicating Lauderhill residents face disproportionate barriers to opportunity and well-being. The median length of living in the US was 46 years (range = 85 years), and the median length of residing at the current zip code is 14.2 years (range = 50). When asked if participants faced COVID challenges, 23 (46%) reported yes. Among these individuals, 14 (52 %) people who faced challenges during the pandemic were Black/African American, and six (23%) were white. Among respondents diagnosed with COVID-19, no difference was found by ethnicity, race, sex, access to a primary physician and insurance availability (Table 4).

**Table 3:** Economic conditions

	Overall
	(N=235)
Education	
< HS Diploma	3 (1.3%)
HS Diploma or GED	6 (2.6%)
More than High School	94 (40.0%)
Missing	132 (56.2%)
Working Fulltime	
Unemployed	22 (9.4%)
Full-time work	62 (26.4%)
Unknown	6 (2.6%)
Missing	145 (61.7%)
Unemployed Reason	
Disabled	3 (1.3%)
Retired	18 (7.7%)
Student	1 (0.4%)
Hours Worked	
<20 hours a week	0 (0%)
20-34 hours a week	9 (3.8%)
35-59 hours a week	50 (21.3%)
60 hours or more a week	8 (3.4%)
Missing	168 (71.5%)
Number of Jobs Worked	
1 job	56 (23.8%)
2 jobs	10 (4.3%)
3 or more jobs	3 (1.3%)
Missing	166 (70.6%)
Length Living in the USA	
Mean (SD)	45.4 (18.9)
Median [Min, Max]	46.0 [0.00, 85.0]
Missing	41 (17.4%)
Length at Current Zip Code	16.2 (12.8)
Mean (SD)	14.5 [0.00, 50.0]
Median [Min, Max]	31 (13.2%)
Missing	-

**Table 4:** COVID-19 diagnosis and demographic characteristics

	No	Yes	Overall	
	(N=94)	(N=10)	(N=235)	P-Value
Ethnicity				
Not Hispanic or Latino	84 (89.4%)	9 (90.0%)	102 (43.4%)	0.999
Hispanic or Latino	8 (8.5%)	0 (0%)	11 (4.7%)	
Missing	2 (2.1%)	1 (10.0%)	122 (51.9%)	
Race				0.999
Multiple Races	8 (8.5%)	3 (30.0%)	12 (5.1%)	
African American	32 (34.0%)	3 (30.0%)	39 (16.6%)	
Afro-Caribbean	10 (10.6%)	1 (10.0%)	13 (5.5%)	
American Indian	1 (1.1%)	0 (0%)	1 (0.4%)	
Asian	2 (2.1%)	0 (0%)	2 (0.9%)	
West Indian	9 (9.6%)	1 (10.0%)	10 (4.3%)	
White	24 (25.5%)	1 (10.0%)	29 (12.3%)	
Other	3 (3.2%)	1 (10.0%)	4 (1.7%)	
Missing	5 (5.3%)	0 (0%)	125 (53.2%)	
Sex				0.386
Female	75 (79.8%)	6 (60.0%)	157 (66.8%)	
Male	17 (18.1%)	3 (30.0%)	52 (22.1%)	
Other	1 (1.1%)	0 (0%)	5 (2.1%)	
Missing	1 (1.1%)	1 (10.0%)	21 (8.9%)	
Primary Care Doctor				0.103
Yes	16 (17.0%)	4 (40.0%)	22 (9.4%)	
No	76 (80.9%)	6 (60.0%)	91 (38.7%)	
Missing	2 (2.1%)	0 (0%)	122 (51.9%)	
Insurance				0.191
Yes	41 (43.6%)	7 (70.0%)	51 (21.7%)	
No	46 (48.9%)	3 (30.0%)	50 (21.3%)	
Missing	7 (7.4%)	0 (0%)	134 (57.0%)	

Health

Twenty-three (9.8%) of respondents were previously diagnosed with cancer, with breast cancer being the most common (5%). The median BMI was 28.4 (IQR = 6.83), seven (38%) respondents were of normal weight, two (14%) obese, six (33%) overweight, and three (15%) underweight. Nineteen (8%) of all respondents had diabetes, while two (1%) of women had gestational diabetes. Additionally, 42 (18%) are smokers, 10 (4%) report chronic back pain, 30 (13%) lack energy, 42 (18%) report high blood pressure and 85 (36%) are physically active (Table 5). 

**Table 5:** Health conditions

	Overall (N=235)
Cancer	
No	170 (72.3%)
Yes	23 (9.8%)
Missing	42 (17.9%)
Smoker	
No	159 (67.7%)
Yes	42 (17.9%)
Missing	34 (14.5%)
Lack of Energy	
No	83 (35.3%)
Yes	30 (12.8%)
Missing	122 (51.9%)
Back Pain	
No	102 (43.4%)
Yes	10 (4.3%)
Missing	123 (52.3%)
Diabetes	
No	96 (40.9%)
Yes	19 (8.1%)
Missing	120 (51.1%)
Gestational Diabetes	
No	89 (37.9%)
Yes	2 (0.9%)
Missing	144 (61.3%)
HBP	
No	72 (30.6%)
Yes	42 (17.9%)
Missing	121 (51.5%)
Physically Active	
No	30 (12.8%)
Yes	85 (36.2%)
Missing	120 (51.1%)
BMI	
Mean (SD)	28.8 (5.34)
Median [Min, Max]	28.4 [21.7, 41.4]
Missing	217 (92.3%)

Social Determinants

The median income was 79,000 (IQR=66,750), 104 (44%) have housing, and of those with housing, nine (9%) were worried about losing their housing. Additionally, only one (0.4%) respondent was a refugee, nobody had spent time in jail, 90 (38%) felt emotionally safe, and one person (0.4%) was afraid of a partner or ex-partner. 

Of 235 participants, six (3%) reported being unable to access required medication, while three (2%) people reported being unable to get childcare, clothing, food, utilities, phone, and other services. Regarding insurance, 55 (23%) of individuals reported having private insurance, 18 (8%) had Medicare, and eight (3%) were uninsured. Three individuals (1%) reported having a lack of transportation that prevented them from attending medical appointments, 18 (8%) reported talking with family or family and friends, and 56 (24%) reported speaking to their family or friends more than five times a week. Last, over 48 (20%) reported moderate or higher stress levels (Table 6). Furthermore, these rates surpass county-level benchmarks, indicating concentrated disparities among Lauderhill’s most vulnerable populations. 

**Table 6:** Social conditions (percentages calculated from valid responses per item (denominator varies) Missing responses are shown separately.

	Overall (N=235)
Type of Insurance	
Private	55 (23.4%)
Medicare	18 (7.7%)
Medicaid	8 (3.4%)
Other	7 (3.0%)
Uninsured	8 (3.4%)
Missing	139 (59.1%)
Lack of Transportation	
No	232 (98.7%)
Yes	3 (1.3%)
Who do You Talk With	
Family	8 (3.4%)
Family and Friends	10 (4.3%)
Friends	3 (1.3%)
Parents	2 (0.9%)
Other	0 (0%)
Missing	212 (90.2%)
How Often Do You Talk	
<1 Time a Week	9 (3.8%)
1-2 Times a Week	18 (7.7%)
3-5 Times a Week	15 (6.4%)
5+ Times a Week	56 (23.8%)
Missing	137 (58.3%)
Stress Level	
None	17 (7.2%)
A Little Bit	31 (13.2%)
Moderate	31 (13.2%)
Quite a Bit	7 (3.0%)
Very Much	10 (4.3%)
Missing	139 (59.1%)

The City of Lauderhill Police Department’s Records Management System captured 9,065 total incident reports from April 15, 2020, to April 15, 2021. Overall, theft and burglary topped the incidents that transpired during the period, followed by aggravated assault/battery and, to a lesser extent, motor vehicle theft, as well as residential and business burglary. Homicides occurred to the lowest extent compared with overall theft and burglary. 

The City of Lauderhill Fire Department’s database captured 7,264 total transported medical calls from April 15, 2020, to April 15, 2021. The top five service calls include trauma (2,444), neurological emergencies (1,118), other/general weaknesses (788), gastrointestinal emergencies (784), and respiratory emergencies (628). Patients were transported to 5 area hospitals, including Florida Medical Center (3,302), Plantation General Hospital (1,713), University Hospital (693), Broward General (396), and Westside Hospital (195). 

Ninety-five percent of the cases transported by EMS were adults (6889), and five percent were children 17 and under (374). Sixty-nine percent (5012) were Black or African American, 25% were White (1816), and 5% were Hispanic (363). More women (53%, N=3850) were transported than men (47%, N=3414). 

Qualitative data results

The qualitative data obtained from focus groups contextualizes the quantitative data and helps interpret the community’s perspective. In Table 7, participants were asked broader questions about the positive and negative factors of the city, as well as about what they would change. In Table 8, each of the eight focus groups represented brought up specific challenges pertaining to their group. For example, one resident shared, "People feel disconnected from clinics-they don’t speak our language or understand our issues." Another remarked, "The kids have no safe space. That’s why violence keeps rising.” A participant emphasized, "We see violence because kids don’t have mentors or jobs, just empty parks and police." This reflects the complex intersection between community resources and public safety. In Table 9, focus groups outlined challenges present in the City of Lauderhill and suggestions for improvement in terms of the Social Determinants of Health. Overall, the focus groups contributed to a better understanding of the community alongside the quantitative data in order to fully grasp the needs of the City of Lauderhill.

**Table 7:** Resident perspectives on living and working in Lauderhill: a dual view of positives and challenges

Resident perceptions	
Top 5 Positive Reasons Participants were Satisfied with Living and/or Working in the City of Lauderhill	Diversity / multicultural melting pot
	Central geographical location
	Sense of community / authentic, caring, resilient, supportive people
	Small city with opportunities to get involved
	City Commission and staff are accessible
Top 5 Challenges Participants Believe that the City of Lauderhill is Facing	Crime / police presence
	Taxes are too high
	Negative public perception and reputation
	Cleanliness / appearance of the city
	Information dissemination / not aware of resources in Lauderhill
If Participants Could Improve One Thing About the Community	Crime
	Taxes
	Beautification
	Community Connection
	Information Dissemination

**Table 8:** Perceived challenges in Lauderhill: insights from diverse focus groups

Perception of challenges faced by focus groups	
Business Focus Group	Access to capital
	Growing homelessness issues
	Lack of business involvement in city events
Resident Focus Group	High percentage of rental units
	School conditions
	Cleanliness vs. perception of safety
	Homeless population increasing
Faith Leaders’ Focus Group	Lack of opportunity for collaborations
	Limited youth transportation
Nonprofit Focus Group	Job availability
	Coordination between government officials
Healthcare Focus Group	Lack of trust in local healthcare services
	Lack of access to/use of healthcare within the city
Education Focus Group	Mental health and trauma
	Effects of COVID on academic training
Senior Focus Group	Crime
	Sanitation and cleanliness
	Communication of city resources for seniors
Youth Focus Group	Lack of preparation with life skills for after high school
	Violent crime

**Table 9:** Social determinants of health - participant challenges and suggestions

Social determinant of health	Challenges presented by participants	Suggestions for improvement by participants
Health	Mental health	Increase mental health programs for children and adults
	Access to affordable and quality healthcare	Increase access to healthcare
	Lack of prevention and nutrition initiatives	Health education initiatives
	Well-being of children in neighborhoods with high violent crimes	
Built Environment	Crime	Initiate beautification projects
	Access to affordable, healthy food	Improve resident safety
	Cleanliness	
Education	Poorly-rated schools	Increase enrichment programs in underperforming schools
	Families sending children to schools outside city	Invest in youth programs
	Limited youth programs	
Economic Stability	High taxes	Lower taxes
	Poor access to capital	Address housing and homeownership concerns
	Housing issues	
	Rising homeless population	
Community Context and Communication	Lack of community engagement	Improve local government communication and engagement
	Lack of shared vision for the city	

## Discussion

Our findings reflect and reinforce the interconnectedness of social vulnerability and poor health outcomes, patterns consistent with national SDOH frameworks like Healthy People 2030 and CDC SVI indicators. The identified disparities reflect structural determinants such as housing and access to care rather than isolated behaviors, and shape community well-being. In this analysis of SDOH within the City of Lauderhill, an evaluation was conducted on the overall social vulnerability index, socioeconomic status, housing, and transportation access. The correlation between Lauderhill’s high SVI and elevated disease prevalence (e.g., asthma, hypertension) mirrors documented national trends linking SDOH to health disparities. 

The initial assessment revealed that there remain some barriers to residents thriving within the city due to issues tied to social, health, economic, and quality of life outcomes and risks. Social and economic status vulnerabilities are significant problems within the city, most pronounced in the youth and east regions, particularly when compared to Broward County as a whole. Meanwhile, housing and transportation vulnerability appear to be less of an issue within the city as compared to the overall county. However, there are several regions within the southeast region that are more greatly impacted. Understanding the underlying factors tied to higher rates of social vulnerability and economic status vulnerability for the City of Lauderhill compared to the overall county is warranted and much needed to improve health outcomes, well-being, and quality of life of the city’s residents. 

The rate of overall social vulnerability within the City of Lauderhill is approximately 31% higher than in Broward County, which is significant for most regions within the city. Only a few regions within the southwest region of the city have rates in the lower quintiles. Similar to rates of diabetes, asthma, poor physical and mental health, and smoking, higher rates of overall social vulnerability appear to be most pronounced in the southeast region of the city. 

Socioeconomic status vulnerability appears to be a more significant problem for this city than overall social vulnerability. It is also a more significant problem as compared to Broward County overall. The City of Lauderhill has an average overall socioeconomic status vulnerability of approximately 73% higher than the county, with the highest rates in the southeast region of the city, similar to overall social vulnerability. 

Housing and transportation vulnerability appear to be less of an issue within the City of Lauderhill as compared to Broward County overall. The overall housing and transportation vulnerability rate is 3% lower than the county’s. Still, there are several regions across the city, especially in the south and east regions of the city limits, that are in higher quintiles. Homeless data was obtained during a 10-day Point in Time (PIT) count. There was an 18-fold increase in the number of homeless individuals in the City of Lauderhill in 2021 as compared to 2020. 

Participants in all eight focus groups outlined challenges related to each SDOH impact and communication. For healthcare, mental health, well-being of children, access to healthcare, lack of prevention and nutrition initiatives, and distrust of the healthcare system arose as key issues among participants. Key educational challenges in the City of Lauderhill include the fact that several public schools have lower performance ratings compared to neighboring districts, leading many families to enroll their children in schools outside the city. Additionally, the limited availability of youth enrichment and after-school programs within Lauderhill constrains opportunities for children and adolescents to engage in structured developmental activities. As noted above, high taxes, poor access to capital, a rising homeless population, and housing issues were concerns that arose regarding economic stability. As for neighborhood and built environment, safety and environmental factors arose as the biggest concerns. Creating sustainable community gardens is one example of an initiative that can address community needs. According to Litt et al., community gardens not only foster social cohesion but have also been linked to increased fruit/vegetable (FV) intake. Community gardeners consumed FV ~5.7 times/day versus ~3.9 for non-gardeners [10]. 

Screening for social determinants of health can allow providers to obtain a better understanding of factors affecting patient health in a population, as well as underlying needs. Initial assessment demonstrates an unmet need to address the lack of health insurance and routine doctor visits within the city that is greater than Broward County overall. In addition, health outcomes, especially asthma, diabetes, stroke, high blood pressure, and obesity prevalence, as well as rates of physical and mental health and smoking rates, are much higher in the city as compared to Broward County, which is the highest and most pronounced in the southeast region of the city. Other factors, including coronary artery disease, health insurance access, and lack of routine doctor’s visits, are higher than the overall county but are not as high as other health outcomes and preventative strategies, and are more widespread (as opposed to concentrated in the south and east regions of the city). Meanwhile, rates of binge drinking are significantly lower than in the county. 

The COVID-19 pandemic further exacerbated many of the challenges identified in this community. During the pandemic, access to routine healthcare visits declined due to clinic closures, reduced in-person services, and fear of exposure, leading to delayed diagnoses and interruptions in chronic disease management. Social determinants of health, such as housing instability, food insecurity, and unemployment, worsened as many families experienced job loss and economic hardship. Mental health conditions, including anxiety, depression, and social isolation, became more prevalent, particularly in vulnerable populations. These pandemic-related stressors intensified existing disparities in conditions such as diabetes, hypertension, obesity, and asthma, and widened the gap between this community and Broward County overall. Recognizing the impact of COVID-19 is essential for contextualizing current health disparities and planning effective recovery and resilience strategies. 

It is important to better understand the underlying causes and contributing factors driving these differences in health indicators compared to the overall county, as this knowledge is essential for developing strategies to improve health outcomes, well-being, and quality of life for the city’s residents. 

Health outcomes

The prevalence of asthma among adults within the City of Lauderhill is a significant issue, which is 30% higher than that of Broward County overall. The prevalence of asthma within the city limits is most concentrated and pronounced in the southeast region of the city. The prevalence of coronary heart disease among adults within the City of Lauderhill is not as significant an issue as Asthma within the city limits, but is approximately 5% higher than that of Broward County overall. The prevalence of coronary artery disease across the city varies, with some regions experiencing a more significant burden. The rate of high blood pressure among adults within the City of Lauderhill is 18.5% higher than in Broward County. The rate across the city varies but appears to be most pronounced in the east and south regions of the city. Meanwhile, most of the city is impacted by this condition. 

Stroke is a significant problem for the City of Lauderhill. The rate of stroke among adults within the City of Lauderhill is 37% higher than in Broward County overall. While the rate across the city varies, it appears to be most pronounced in the east region of the city. 

The prevalence of diabetes among adults within the City of Lauderhill is similarly as significant an issue as Asthma within the city limits, approximately 21% higher than that of Broward County overall. The prevalence of coronary artery disease across the city varies, with regions of more significant burden, especially in the southeast region of the city. Cancer is an issue within the City of Lauderhill, but the disease only has a slightly higher rate of 3.39% compared to Broward County overall. The condition is most pronounced in the north of the city, where you can find an increased cancer burden that is 72% higher as compared with Broward County overall. The higher prevalence of diabetes amongst these individuals may indicate a need for dietary intervention and glucose monitoring. 

Physical health is a greater issue within the City of Lauderhill, compared to Broward County overall, with a poor physical health rate that is 25% higher than the county's. Similar to the prevalence of asthma and diabetes, the highest rates of poor physical health are concentrated and most robust in the southeast region of the city limits. As with poor physical health, mental health is a more significant issue within the City of Lauderhill as compared to Broward County overall, with the poor mental health rate 31% higher than the county. Similar to the prevalence of asthma and diabetes as well as the rate of poor physical health, the highest rates of poor physical health are concentrated and most robust in the southeast region of the city limits. 

Binge drinking within the City of Lauderhill is not a significant issue as compared to Broward County overall, with binge drinking within the city approximately 13% lower than in the overall county. The behavior is relatively low throughout most of the city, with only very small pockets of elevated binge drinking regions within the city limits. Smoking is one of the most significant issues within the City of Lauderhill, with the adult smoking rate approximately 40% higher than in Broward County. The most pronounced rates of smoking are concentrated in the south and east regions of the city, which is similar to rates of asthma and diabetes, as well as poor physical health. The incidence of cigarette smoking among residents was high, indicating a strong need for smoking cessation initiatives. 

Obesity is a greater issue within the City of Lauderhill than in Broward County overall. The rate of adult obesity within the city is 22% higher as compared to the overall county, and the most pronounced and concentrated rates appear in the south and east regions of the city limits. Mental health, well-being of children, access to healthcare, lack of prevention and nutrition initiatives, and distrust of the healthcare system also arose as key issues. 

Health insurance access within the City of Lauderhill is a greater issue than in Broward County, with the percentage range approximately 13% higher among those 18 to 64 years of age within the city limits as compared to the county overall. This limited health insurance access rate is concentrated and most pronounced in the southeast region of the city limits. In the year before our study was completed, doctor visits for routine check-ups among adults within the City of Lauderhill were approximately 3% higher than in Broward County. Most regions within the city have higher rates than the overall county, with only a few areas demonstrating lower rates than the county. 

Prevention

To combat these issues of inequality, here we propose several solutions. Firstly, during the initial insurance registration process, the presence of on-site staff to assist can greatly facilitate the enrollment process and ensure that individuals have access to necessary coverage. This approach minimizes barriers and streamlines the insurance sign-up process, enabling more individuals to obtain essential healthcare services. 

Secondly, patient navigators can be instrumental in guiding patients through the complex healthcare system, providing support, and ensuring they receive appropriate care. Patient navigators can offer personalized assistance, particularly to underserved and underrepresented populations, addressing cultural, linguistic, and logistical challenges that may impede healthcare access. In fact, in randomized trials, integrating social needs screening with in-person navigation led to significant reductions in family-reported social risks and improvements in pediatric health within four months [11]. Furthermore, integrating community health workers (CHWs) selected from the populations of focus can significantly improve health education efforts. These CHWs, familiar with their communities' cultural nuances and languages, can effectively deliver culturally tailored health education, bridging the gap between healthcare providers and residents. 

Further, implementing culturally tailored health education materials and programs within healthcare facilities, both in print and through visual storytelling, can engage and empower individuals in their own healthcare journeys. Through presenting information in a culturally sensitive manner, these initiatives promote health literacy, encourage preventive measures, and foster stronger connections between healthcare providers and the community they serve. 

Recommendations

The City of Lauderhill community needs assessment identified health outcomes to be addressed, the first step toward health equity in the CoL. The social determinants of health provide a framework for advancement and organization for potential multifaceted interventions. It is critical to have a throughline within future health programs of culturally informed and community-based recommendations, as the success of the initiatives, and therefore, the improved health of participants, is influenced by such factors. Such is the goal of programs like NIH’s Community Partnerships to Advance Science for Society (ComPASS), which emphasizes the collaboration between researchers and communities [12]. 

For the CoL, programs focused on the health concerns described above, specifically physical health, obesity, diabetes, stroke, and asthma, would target the issues identified in the community needs assessment. Further examination of each specific concern, including assessing community knowledge and perceived need, would be the immediate next steps before initiative implementation. 

Addressing nutrition would include examining food access, including affordability, accessibility, cultural alignment, and quality. Community engagement would help along each step of the intervention, which is important for community acceptance and success. The partnership would also provide insight into eating and shopping habits, opening the possibility for health literacy initiatives such as the Caribbean Diaspora Healthy Nutrition Outreach Project [13] and grassroots community health programs such as “In the Kitchen with Dr. H.” Addressing the city’s nutritional challenges will require targeted strategies to improve access to healthy foods and reduce diet-related health disparities. Recommended approaches include expanding mobile food pantry services and developing urban community gardens to increase the availability of fresh produce in underserved neighborhoods. Schools and faith-based organizations could serve as hubs for nutrition education, promoting healthier eating habits among children and families. Partnerships with local farmers’ markets could also be explored to provide subsidized produce vouchers for low-income households. These initiatives could be implemented through collaboration between community-based organizations, municipal agencies, and health systems, with financing potentially drawn from federal programs such as SNAP and WIC, local government funding, and philanthropic support. Strong community social capital has been shown to mitigate food insecurity and strengthen collective resilience during public health crises, reinforcing our focus on resident-driven partnerships [14]. Prioritizing these nutrition-focused interventions would help mitigate food insecurity, improve dietary quality, and address the higher burden of obesity, diabetes, and hypertension in the city. 

The infrastructure needed for food access consists of the presence of shops and transportation availability, including sidewalks and biking lanes. This infrastructure additionally lends itself to the space and safety needed for physical activity, another significant component affecting the target health concerns. As a foundation of public health, prevention would be an essential and primary component of recommended programs. Upstream social determinants of health, including those for children and families, show promise for community and population-level health disparities [15]. 

Limitations

This small sample size limits this study. Most data were self-reported by participants. Notably, the primary limitation of this study is the substantial amount of missing data across several survey variables, which limits interpretation and generalizability. Additionally, while our qualitative approach prioritized inclusivity and contextual richness, it also introduced limitations related to reproducibility and the potential for interpretive bias. The absence of formal coding may affect the perceived transparency of theme development, and subtle insights voiced less frequently could be underrepresented. 

Participants, for the most part, were educated, in good economic standing, and had private insurance, as gathered by the completed PRAPARE survey. Additionally, due to variability in the study population, findings may not fully represent the Lauderhill population. Future studies warrant incorporating a larger sample to include a more diverse and representative sample. Moreover, participants were self-selected, which may introduce selection bias. As such, generalizability beyond Lauderhill, or even beyond survey participants within the city, must be made with caution. 

Further investigation into age, gender, race, cultural variables, and geographic factors affecting these health indicators is warranted. Due to the widespread nature of these conditions, targeted approaches are likely needed to address these issues across the city. Overall, the factors driving differences in health indicators between the city and the county are essential for improving health outcomes, well-being, and quality of life for the city’s residents [16]. In addition, community health education, health literacy training, and assessing sources of physical activity and healthy foods are essential next steps. 

## Conclusions

This anonymous questionnaire, distributed to participants aged 18 years or older, and who either resided and/or worked in the City of Lauderhill, highlighted the demographic, economic, and health status within the city limits. Findings indicate critical SDOH priorities for Lauderhill: access to mental health services, stable housing, food security, and employment opportunities. The needs assessment demonstrated a diverse demographic characterized by hard-working individuals, with limited issues tied to access to their needs being met, and limited housing concerns and transportation issues. Given the overarching health metrics outlined in the BRHPC data report as aligned with the community survey data, attention to smoking cessation initiatives, mental health, overall health, wellness, disease prevention, and physical activity programs are essential and warranted. 

The mixed-methods evaluation of the social determinants of health in the CoL through multifaceted approaches built a more comprehensive picture of the city and residents’ overall health, meeting this study's purpose and overarching goal. In addition, actionable SDOH needs were identified, particularly around access to care, mental health, food insecurity, and housing, which have informed Lauderhill’s integrated public-private response model. Findings support the implementation of data-driven health equity strategies in municipalities with similar demographic profiles. Fostering strong community social capital enhances food security and strengthens collective resilience, emphasizing the importance of resident-driven partnerships. The elements identified through this work hold the potential to inform decisions and set priorities in addressing noted health disparities and continuing advancement toward a prosperous community health outlook. The key feature was the collaboration across public and private stakeholders and individuals to inform and develop recommendations. Overall, understanding and acting upon the underlying factors tied to higher rates of overall social vulnerability and economic status vulnerability of the CoL is needed to improve health outcomes, well-being, and quality of life of the city’s residents. An adaptable, open partnership between an anchor academic institution, city residents, and local businesses is essential to achieve health equity. Municipalities with similar demographic and structural challenges can leverage these findings to develop integrated, resident-informed health equity policies grounded in lived experience. 

## Appendices

**Table 10:** Community health questionnaire

Question	Response								
Are you 18 or older?	Yes	No							
Since this survey is in English, do you read, speak and understand English?	Yes	No							
Have you already completed this survey?	Yes	No	Not sure						
How do you describe yourself?	Male	Female	Trans Male/Trans Man	Trans Female/Trans Woman	Genderqueer	Gender Nonconforming	Different Identity	I choose not to answer this question.	
What sex were you assigned at birth, such as on an original birth certificate?	Male	Female							
What zip code do you live in?	________________								
Length of time living at the current zip code:	________________								
Length of time living in the USA:	________________								
What is your country of birth?	________________								
How long have you lived in the City of Lauderhill?	________________								
If you do not live in the City of Lauderhill, what city, county and state do you live in?	________________								
What is your primary language?	English	Spanish	Creole	Other					
Please list any languages that you speak.	________________								
Health History - Tobacco, Smoking									
Do you or did you ever smoke cigars, cigarettes, or vape?	Yes	No							
Please tell us what you smoke(d). Check all that apply:	Cigarette smoking	Smoking tobacco products other than cigarettes	Smokeless tobacco products	Tobacco, not otherwise specified.					
How many total years have you used any tobacco product?	________________								
Cancer History									
Have you ever been diagnosed with cancer?	Yes	No							
What type of cancer have you been diagnosed with?	Breast	Lung	Prostate	Colorectal	Brain	Bone	Other		
What other type(s) of cancer have you been diagnosed with?	________________								
Zip code at time of cancer diagnosis:	________________								
Length of time living in zip code at time of cancer diagnosis:	________________								
Payer at diagnosis (no insurance, self-pay, insurance type, etc.):	________________								
Are you now cancer-free?	Yes	No							
Personal Cancer Screening History. Record your screening history.	________________								
For Female Participants:									
Breast--Clinical Breast Exam. When was the most recent?	________________								
Breast--Clinical Breast Exam. How often?	________________								
Breast--Clinical Breast Exam. Age started?	________________								
Breast--Clinical Breast Exam. Comments.	________________								
Breast--Mammogram. Most recent?	________________								
Breast--Mammogram. How often?	________________								
Breast--Mammogram. Age started?	________________								
Breast--Mammogram. Comments.	________________								
Cervical--PAP Smear. Most recent?	________________								
Cervical--PAP Smear. How often?	________________								
Cervical--PAP Smear. Age started?	________________								
Cervical--PAP Smear. Comments.	________________								
Cervical--Pelvic Exam. Most recent?	________________								
Cervical--Pelvic Exam. How often?	________________								
Cervical--Pelvic Exam. Age started?	________________								
Cervical--Pelvic Exam. Comments.	________________								
For Male Participants:									
Prostate--Digital Rectal Exam. Most recent?	________________								
Prostate--Digital Rectal Exam. How often?	________________								
Prostate--Digital Rectal Exam. Age started?	________________								
Prostate--Digital Rectal Exam. Comments.	________________								
Prostate--PSA Blood Test. Most recent?	________________								
Prostate--PSA Blood Test. How often?	________________								
Prostate--PSA Blood Test. Age started?	________________								
Prostate--PSA Blood Test. Comments.	________________								
Cancer Screening History:									
Colorectal--Colonoscopy. Most recent?	________________								
Colorectal--Colonoscopy. How often?	________________								
Colorectal--Colonoscopy. Age started?	________________								
Colorectal--Colonoscopy. Comments.	________________								
Colorectal--Sigmoidoscopy. Most recent?	________________								
Colorectal--Sigmoidoscopy. How often?	________________								
Colorectal--Sigmoidoscopy. Age started?	________________								
Colorectal--Sigmoidoscopy. Comments.	________________								
Colorectal--At home stool test. Most recent?	________________								
Colorectal--At home stool test. How often?	________________								
Colorectal--At home stool test. Age started?	________________								
Colorectal--At home stool test. Comments.	________________								
Colorectal--Other. What and most recent?	________________								
Colorectal--Other. How often?	________________								
Colorectal--Other. Age started?	________________								
Colorectal--Other. Comments.	________________								
Personal Cancer History:									
Have you ever been diagnosed with uterine cancer?	Yes	No							
What was your age at the time of your uterine cancer diagnosis?		________________								
Please comment on your uterine cancer.	________________								
Have you ever been diagnosed with ovarian cancer?	Yes	No							
What was your age at the time of your ovarian cancer diagnosis?	________________								
Please comment on your ovarian cancer.	________________								
Have you ever been diagnosed with colorectal cancer?	Yes	No							
What was your age at the time of your colorectal cancer diagnosis?	________________								
Please comment on your colorectal cancer.	________________								
Have you ever been diagnosed with breast cancer?	Yes	No							
What was your age at the time of your breast cancer diagnosis?	________________								
Please comment on your breast cancer.	________________								
Have you ever been diagnosed with prostate cancer?	Yes	No							
What was your age at the time of your prostate cancer diagnosis?	________________								
Please comment on your prostate cancer.	________________								
Have you ever been diagnosed with cervical cancer?	Yes	No							
What was your age at the time of your cervical cancer diagnosis?	________________								
Please comment on your cervical cancer.	________________								
Have you ever been diagnosed with another type of cancer?	Yes	No							
What other type of cancer were you diagnosed with?	________________								
What was your age at the time of your other cancer diagnosis?	________________								
Please comment on your other cancer.	________________								
Personal Symtpoms:									
Have you had a change in bowel habits, such as diarrhea, constipation, or narrowing of the stool, that lasts for more than a few days?	Yes	No							
Comments on change in bowel habits.	________________								
Rectal bleeding?	Yes	No							
Comments on rectal bleeding.	________________								
Dark stools, or blood in the stool (poop)?	Yes	No							
Comments on dark stools, or blood in the stool (poop).	________________								
Cramping or abdominal pain?	Yes	No							
Comments on cramping or abdominal pain.	________________								
Weight loss?	Yes	No							
Comments on weight loss.	________________								
Fatigue, lack of energy?	Yes	No							
Comments on fatigue, lack of energy.	________________								
New back pain?	Yes	No							
Comments on new back pain.	________________								
Changes in appearance of one or both nipples?	Yes	No							
Comments on changes in appearance of one or both nipples.	________________								
Nipple discharge?	Yes	No							
Comments on nipple discharge.	________________								
General pain in/on any part of the breast?	Yes	No							
Comments on general pain in/on any part of the breast.	________________								
Irritated or itchy breasts?	Yes	No							
Comments on irritated or itchy breasts.	________________								
Change in breast color, size, shape, or touch?	Yes	No							
Comments on change in breast color, size, shape, or touch.	________________								
Peeling or flaking of the nipple skin?	Yes	No							
Comments on peeling or flaking of the nipple skin.	________________								
For Females									
Vaginal bleeding?	Yes	No							
Comments on vaginal bleeding.	________________								
Unusual vaginal discharge or odor?	Yes	No							
Comments on unusual vaginal discharge or odor.	________________								
Pelvic pain?	Yes	No							
Comments on pelvic pain.	________________								
Family Cancer History									
Uterine - Relation	________________								
Uterine - Age at diagnosis	________________								
Uterine - Age at death	________________								
Uterine - Comments	________________								
Ovarian - Relation	________________								
Ovarian - Age at diagnosis	________________								
Ovarian - Age at death	________________								
Ovarian - Comments	________________								
Colorectal - Relation	________________								
Colorectal - Age at diagnosis	________________								
Colorectal - Comments	________________								
Breast - Relation	________________								
Breast - Age at diagnosis	________________								
Breast - Age at death	________________								
Breast - Comments	________________								
Prostate - Relation	________________								
Prostate - Age at diagnosis	________________								
Prostate - Age at death	________________								
Prostate - Comments	________________								
Cervical - Relation	________________								
Cervical - Age at diagnosis	________________								
Cervical - Age at death	________________								
Cervical - Comments	________________								
Other cancer - Type and relation	________________								
Other cancer - Age at diagnosis	________________								
Other cancer - Age at death	________________								
Other cancer - Comments	________________								
Diabetes									
Do you have diabetes?	Yes	No							
Were you ever told that you have high blood sugar levels, prediabetes, impaired glucose tolerance or that based on your bloodwork you are at risk for developing diabetes?	Yes	No	Not sure						
Do you have Type 1 (insulin only) or Type 2?	Type 1	Type 2	Not sure						
If you are a woman, have you ever been diagnosied with gestational diabetes	Yes	No							
Do you have a mother, father, sister or brother with diabetes?	Yes	No							
Have you ever been diagnosed with high blood pressure?	Yes	No							
Are you physically active?	Yes	No							
How many feet tall are you? (Please just give the number of feet; inches)	________________								
How many inches?	________________								
Do you know approximately how much you weigh?	Yes	No							
Please provide your weight and include whether the weight is measured in lbs or kgs	________________								
Assessment of Care for Chronic Conditions - Diabetes Care									
Asked for my ideas when we made a treatment plan	Almost Never	Generally Not	Sometimes	Most of the Time	Almost Always				
Given choices about treatment to think about	Almost Never	Generally Not	Sometimes	Most of the Time	Almost Always				
Given a written list of the things I should do to improve my health	Almost Never	Generally Not	Sometimes	Most of the Time	Almost Always				
Satisfied that my care was well organized	Almost Never	Generally Not	Sometimes	Most of the Time	Almost Always				
Shown how what I did to take care of my illness influenced my condition	Almost Never	Generally Not	Sometimes	Most of the Time	Almost Always				
Asked to talk about my goals in caring for my illness	Almost Never	Generally Not	Sometimes	Most of the Time	Almost Always				
Helped to set specific goals to improve my eating or exercise	Almost Never	Generally Not	Sometimes	Most of the Time	Almost Always				
Given a copy of my treatment plan	Almost Never	Generally Not	Sometimes	Most of the Time	Almost Always				
Encouraged to go to a specific group or class to help me cope with my chronic illness	Almost Never	Generally Not	Sometimes	Most of the Time	Almost Always				
Asked questions, either directly or on a survey, about my health habits	Almost Never	Generally Not	Sometimes	Most of the Time	Almost Always				
Sure that my doctor or nurse thought about my values and my traditions when they recommended treatments to me	Almost Never	Generally Not	Sometimes	Most of the Time	Almost Always				
Helped to make a treatment plan that I could do in my daily life	Almost Never	Generally Not	Sometimes	Most of the Time	Almost Always				
Helped to plan ahead so I could take care of my illness even in hard times	Almost Never	Generally Not	Sometimes	Most of the Time	Almost Always				
Asked how my chronic illness affects my life	Almost Never	Generally Not	Sometimes	Most of the Time	Almost Always				
Contacted after a visit to see how things were going	Almost Never	Generally Not	Sometimes	Most of the Time	Almost Always				
Encouraged to attend programs in the community that could help me	Almost Never	Generally Not	Sometimes	Most of the Time	Almost Always				
Referred to a dietitian, health educator, or counselor	Almost Never	Generally Not	Sometimes	Most of the Time	Almost Always				
Told how my visits with other types of doctors, like the eye doctor or surgeon, helped my treatment	Almost Never	Generally Not	Sometimes	Most of the Time	Almost Always				
Asked how many visits with other doctors were going	Almost Never	Generally Not	Sometimes	Most of the Time	Almost Always				
Asked what I would like to discuss about my illness at that visit	Almost Never	Generally Not	Sometimes	Most of the Time	Almost Always				
Asked how my work, family, or social situation related to taking	Almost Never	Generally Not	Sometimes	Most of the Time	Almost Always				
Helped to make plans for how to get support from my friends, family or community	Almost Never	Generally Not	Sometimes	Most of the Time	Almost Always				
Told how important the things I do to take care of my illness (ex. Exercise) were to my health	Almost Never	Generally Not	Sometimes	Most of the Time	Almost Always				
Set a goal together with my team for what I could do to manage my condition	Almost Never	Generally Not	Sometimes	Most of the Time	Almost Always				
Given a book or monitoring log In which to record the progress I am making	Almost Never	Generally Not	Sometimes	Most of the Time	Almost Always				
Personal Characteristics									
Is your primary care doctor from the Caribbean?	Yes	No	I do not know						
Are you Hispanic or Latino?	Yes	No	I choose not to answer this question						
Which race(s) are you? Check all that apply	Asian	West Indian	Native Hawaiin	Pacific Islander	Black/African American /Afro-Caribbean	America Indian/ Alaskan Native	White	Other	I choose not to answer this question
What other race(s) are you?	________________								
At any point in the past 2 years, has seasonal or migrant farm work been your or your family's main source of income?	Yes	No	I choose not to answer						
Have you ever been discharged from the armed forces of the United States?	Yes	No	I choose not to answer this question						
What language are you most comfortable speaking?	English	Language other than English	I choose not to answer this question						
What language other than English are you most comfortable speaking?	________________								
Family and Home									
How many family members, including yourself, do you currently live with?	________________								
What is your housing situation today?	I have housing	I do not have housing (staying with others, in a hotel, in a shelter, living outside on the street, on a beach, in a car, or in a park)	I choose not to answer this question						
Are you worried about losing your housing?	Yes	No	I choose not to answer this question						
Money and Resources									
What is the highest level of school that you have finished?	Less than a high school degree	High school diploma or GED	More than high school	I choose not to answer this question					
What is your current work situatiion?	Unemployed and seeking work	Part time or temporary work	Full time work	Otherwise, unemployed but not seeking work (ex. student, retired, disabled, unpaid primary care giver)	I choose not to answer this question				
Please choose what other unemployed but not seeking work category describes your situation	Student	Retired	Disabled	Unpaid primary care giver					
If you are employed or workng, how many jobs do you work?	1 job	2 jobs	3 or more jobs	I choose not to answer this question					
What is your main insurance?	None/uninsured	Medicaid	CHIP Medicaid	Medicare	Other Public Insurance (Not CHIP)	Other Public Insurance (CHIP)	Private insurance	I am not sure	Other
Please descrive what insruance you have	________________								
Do you have insurance through your jon?	Yes	No	I choose not to answer this question						
During the past year, what was the total combined income for you and your family members you live with?	________________								
In the past year, have you or any family members you live with been unable to get any of the following when it was really needed? Check all that apply.	Yes, Food	Yes, Clothing	Yes, Utilities	Yes, Child Care	Yes, Medicine or any health care (medical, dental, mental health, vision)	Yes, Phone	Yes, Other	No, I have not had any problems getting things that I need	I choose not to answer this question
What other item(s) were you or any family members you live with unable to get when they were really needed in the past year?	________________								
Has lack of transportation kept you from medical appointments, meetings, work, or from getting things needed for daily living. (Please check all that apply).	Yes, it has kept me from medical appointments or from getting my medications	Yes, it has kept me from non-medical meetings, appointments, work, or from getting things that I need	No	I choose not to answer this question					
Social and Emotional Health									
How often do you see or talk to people that you care about and feel close to? (Ex. Talking to friends on the phone, visiting friends or family, going to church or club meetings)	Less than once a week	1 or 2 times a week	3 or 5 times a week	More than 5 times a week	I choose not to answer this question				
Stress is when someone feels tense, nervous, anxious, or can't sleep at night because their mind is troubled. How stressed are you?	Not at all	A little bit	Somewhat	Quite a bit	Very much	I choose not to answer this question			
Who are the people or groups you usually see or talk to at these times? Please describe/write youre response:	________________								
In the past year have you spent more than 2 nights in a row in a jail, prison, detention center, or juvenile correctional facility?	Yes	No	I choose not to answer						
What was your release date?	________________								
Are you a refugee?	Yes	No	I choose not to answer this question						
Do you feel physically and emotionally safe where you currently live?	Yes	No	I choose not to answer this question						
In the past year, have you been afraid of your partner or ex-partner?	Yes	No	Unsure	I have not had a partner in the past year	I choose not to answer this question				
COVID-19									
Have you been tested for COVID-19?	Yes	No							
Did you face difficulty getting tested?	________________								
Have you been diagnosed with COVID-19?	Yes	No							
Did you face difficulty getting treated?	Yes	No							
Have you faced challenges during the COVID-19 pandemic?	________________								
Thank you for your participation in the survey. Any questions/ comments?	________________								
